# PSMA-Targeted Radioligand Therapy Beyond the Post-Taxane Setting: A Review of Evidence Across the Prostate Cancer Spectrum

**DOI:** 10.3390/cancers18132161

**Published:** 2026-07-05

**Authors:** Kaiying Wang, Daanesh Huned Hassanbhai, Roxanne Yong Ai Teo, Chloe Shu Hui Ong, Kah Wai Lai, Si Xuan Koo, Wai Loon Yam, Joshua Yi Min Tung

**Affiliations:** 1 Department of Urology, National University Hospital, Singapore 119074, Singapore; 2 Department of Urology, Tan Tock Seng Hospital, Singapore 308433, Singapore; 3 Department of Nuclear Medicine and Molecular Imaging, Singapore General Hospital, Singapore 169608, Singapore; 4 Department of Urology, Changi General Hospital, Singapore 529889, Singapore

**Keywords:** Lu-PSMA-617, radioligand therapy, prostate cancer, metastatic hormone-sensitive prostate cancer, oligometastatic, PSMA, castration-resistant prostate cancer

## Abstract

Lutetium-177-PSMA-617 (Lu-PSMA) radioligand therapy (RLT) is established in metastatic castration-resistant prostate cancer (mCRPC), with regulatory approvals based on the VISION and TheraP trials. Subsequent trials have extended the evidence to taxane-naive mCRPC (PSMAfore) and demonstrated that combining Lu-PSMA with enzalutamide yields a significant overall survival benefit over enzalutamide alone (ENZA-p). However, higher and more homogeneous PSMA expression in treatment-naive disease, combined with lower tumor burden and preserved bone marrow reserve, provides a biological rationale for deploying RLT earlier in the disease course. In metastatic hormone-sensitive prostate cancer (mHSPC), the Phase III PSMAddition trial reported improved radiographic progression-free survival when Lu-PSMA was added to standard androgen deprivation therapy (ADT) plus androgen receptor pathway inhibitor (ARPI), and the Phase II UpFrontPSMA trial demonstrated enhanced biochemical responses with Lu-PSMA induction before docetaxel. In oligometastatic and oligorecurrent disease, the BULLSEYE and LUNAR trials have shown progression-free survival benefits, raising the possibility of deferring androgen deprivation therapy and its associated morbidity. Meanwhile, next-generation radionuclides, including actinium-225 (WARMTH) and the dual beta-Auger emitter terbium-161 (VIOLET), are entering clinical development to address the radiobiological limitations of Lutetium-177. This review synthesizes the evidence for PSMA-targeted radioligand therapy across the prostate cancer disease continuum and discusses patient selection, treatment sequencing, and the access and cost-effectiveness considerations that will shape adoption in earlier disease settings.

## Simple Summary

Prostate cancer is the most commonly diagnosed cancer in men, and while effective treatments exist, many patients eventually develop drug-resistant disease with limited options. A targeted treatment called radioligand therapy works by attaching a radioactive particle to a molecule that homes in on proteins found abundantly on the surface of prostate cancer cells, delivering radiation directly to the tumour while limiting harm to healthy tissue. Initially approved only for men with heavily pre-treated, advanced prostate cancer, recent clinical trials have now tested this therapy at earlier stages of the disease—including in men with hormone-sensitive cancer and in those with only a small number of detectable tumour deposits. This review synthesises the evidence from these trials to inform how and when radioligand therapy might be best used across the full spectrum of prostate cancer, and identifies research priorities to guide future practice and equitable access globally.

## 1. Introduction

Prostate-specific membrane antigen (PSMA) is a type II transmembrane glycoprotein that is overexpressed in the vast majority of prostate cancers, with expression levels increasing with tumor dedifferentiation, metastatic progression, and androgen deprivation [1,2]. This molecular characteristic has been exploited therapeutically through PSMA-targeted radioligand therapy (RLT), in which a radionuclide, most commonly lutetium-177, is conjugated to a small-molecule PSMA ligand, enabling targeted delivery of beta-particle radiation to PSMA-expressing tumor cells while relatively sparing normal tissues [3].

The clinical development of Lutetium-177 (^177^Lu)–PSMA-617 (“Lu-PSMA”) has followed a characteristic trajectory in oncology. Initial evaluation and trial were conducted in the most advanced, treatment-refractory disease setting, with subsequent investigation in progressively earlier stages as safety and efficacy were proven in advanced-disease cohorts. Regulatory approvals by the US Food and Drug Administration and European Medicines Agency in 2022 were based on the VISION trial, which demonstrated overall survival and radiographic progression-free survival benefits in patients with PSMA-positive mCRPC who had progressed after androgen receptor pathway inhibitor (ARPI) therapy and taxane chemotherapy [3]. Since then, clinical investigation has expanded rapidly across the prostate cancer disease continuum.

Several biological and clinical observations support the rationale for deploying Lu-PSMA earlier in prostate cancer treatment. First, PSMA expression is generally higher and more homogeneous in hormone-sensitive disease than in heavily pretreated mCRPC, where lineage plasticity and neuroendocrine transdifferentiation can lead to PSMA-low or PSMA-negative clones [4,5]. Second, patients with earlier-stage disease typically have better bone marrow reserves and performance status, potentially enabling greater treatment intensity and tolerability. Third, the mechanism of action of Lu-PSMA is direct DNA damage via beta-particle radiation, which is distinct from hormonal therapies and chemotherapy, providing a rationale for combination strategies rather than sequential monotherapy.

This review synthesizes the current evidence for PSMA-targeted RLT across the prostate cancer disease continuum. We review the established evidence in mCRPC, highlighting the trajectory from post-taxane to pre-taxane settings and the emergence of combination strategies. We then examine the emerging data in metastatic hormone-sensitive prostate cancer (mHSPC) and oligometastatic disease—both settings where recent Phase II and III clinical investigations have produced evidence that is reshaping current clinical practice. Finally, we survey next-generation PSMA-targeted radionuclides entering clinical development and discuss the practical considerations and unanswered questions that will shape the integration of RLT into earlier lines of prostate cancer therapy.

## 2. Established Evidence in mCRPC

The clinical development of Lu-PSMA in mCRPC has followed a logical progression: from demonstrating efficacy in heavily pretreated, post-taxane disease, to establishing superiority over alternative systemic therapies, to proving benefit in taxane-naive patients, and most recently, to testing combination strategies with hormonal agents. The trials described below trace this trajectory.

### 2.1. Foundational Evidence in Post-Taxane Disease: VISION and TheraP

The VISION trial established Lu-PSMA as a standard-of-care option in mCRPC. This was an international, open-label, Phase III trial that randomized 831 patients with PSMA-positive mCRPC who had received at least one ARPI and one to two taxane regimens to Lu-PSMA (7.4 GBq every 6 weeks for 4–6 cycles) plus protocol-permitted standard care vs. standard care alone [3]. Lu-PSMA significantly prolonged both co-primary endpoints: median imaging-based progression-free survival (8.7 vs. 3.4 months; hazard ratio [HR] 0.40, 99.2% CI 0.29–0.57; *p* < 0.001) and median overall survival (15.3 vs. 11.3 months; HR 0.62, 95% CI 0.52–0.74; *p* < 0.001). Treatment-related adverse events were predominantly low-grade, with dry mouth (38.8%), fatigue (43.1%), and nausea (35.3%) being most common, though grade 3 or higher hematologic toxicity occurred in a significant proportion of patients (anemia, 12.9%; thrombocytopenia, 7.9%; lymphopenia, 7.8%; leukopenia, 2.5%).

The Australian TheraP trial provided complementary evidence in a head-to-head comparison against an active chemotherapy comparator. This randomized Phase II trial compared Lu-PSMA (6.0–8.5 GBq every 6 weeks for up to 6 cycles) with cabazitaxel in 200 patients with mCRPC progressing after docetaxel [6,7]. Lu-PSMA demonstrated a higher PSA response rate (defined as a reduction of at least 50% from baseline, per-treatment-received analysis 66% vs. 44%; difference 23% CI [9–37]; *p* = 0.0016), longer progression-free survival (restricted mean survival time (RMST) 7.1 vs. 5.0 months; difference 2.1 months CI [0.7–3.6]; *p* = 0.005), and a better safety profile with fewer grade 3–4 adverse events (33% vs. 53%). Notably, mature OS data showed no significant difference between arms (RMST 19.1 months vs. 19.6 months; difference -0.5 months CI [−3.7 to 2.7]; *p* = 0.77), though the trial was not powered for OS and the favorable toxicity profile of Lu-PSMA remained a distinguishing advantage. TheraP also demonstrated that patients with high PSMA expression on 68Ga-PSMA-11 PET derived greater benefit—men with SUVmean of 10 or higher had better odds of PSA response (odds ratio [OR] 12.19 [95% CI 3.42–58.76] vs. 2.22 [1.11–4.51]; *p* = 0.039 [8].

### 2.2. Moving Earlier in mCRPC: PSMAfore

With efficacy and a favorable toxicity profile established in the post-taxane setting, the question became whether Lu-PSMA could benefit patients earlier in the mCRPC disease course. The PSMAfore trial addressed this directly. This Phase III trial randomized 468 patients whose disease had progressed on one prior ARPI to either Lu-PSMA (7.4 GBq, up to 6 cycles) or a change of ARPI (abiraterone or enzalutamide) [9]. Lu-PSMA demonstrated superior radiographic progression-free survival (rPFS; 11.6 vs. 5.6 months; HR 0.49, 95% CI 0.39–0.61) along with improved patient-reported quality of life and pain outcomes. However, the final OS analysis revealed no difference by intention-to-treat (24.48 vs. 23.13 months; HR 0.91) [10]. This result was confounded by the high crossover rate: 60.3% of patients in the ARPI-change arm subsequently received Lu-PSMA. Crossover-adjusted analyses using inverse probability of censoring weighting yielded an OS hazard ratio of 0.59 (95% CI 0.38–0.91), suggesting a true survival benefit masked by the trial’s crossover design.

### 2.3. From Sequential Monotherapy to Combination: ENZA-p

While VISION, TheraP, and PSMAfore all evaluated Lu-PSMA as a monotherapy at different points in the mCRPC trajectory, the ENZA-p trial (ANZUP 1901) tested a fundamentally different premise: whether combining RLT with an ARPI could outperform the ARPI alone. This randomized Phase II trial assigned 162 patients with high-risk first-line mCRPC to enzalutamide with or without adaptive-dosed Lu-PSMA (2 or 4 cycles based on PSMA-PET-CT at 12 weeks) [11,12]. The combination significantly improved PSA progression-free survival (13.0 vs. 7.8 months; HR 0.43, *p* < 0.0001) and, importantly, demonstrated a significant OS benefit at mature follow-up (34 vs. 26 months; HR 0.55, 95% CI 0.36–0.84; *p* = 0.0053), making ENZA-p the first Lu-PSMA trial to demonstrate a significant survival advantage over an active systemic therapy alone, though this was a secondary endpoint. The adaptive dosing strategy guided by early PSMA-PET response also provided proof of concept for biomarker-directed treatment intensification. The combination principle of concurrent RLT plus hormonal therapy established by ENZA-p has been adopted in the design of later trials investigating Lu-PSMA in mHSPC.

## 3. Lu-PSMA in Metastatic Hormone-Sensitive Prostate Cancer

### 3.1. Biological Rationale for Early Intervention

The rationale for deploying Lu-PSMA in mHSPC extends beyond the general principle of early disease treatment. Androgen receptor blockade upregulates PSMA expression in the short term, creating a synergistic opportunity whereby the established backbone of mHSPC treatment enhances the target for radioligand therapy. Furthermore, patients with newly diagnosed mHSPC generally have superior bone marrow reserve and organ function compared with those who have received multiple prior lines of therapy, potentially enabling better tolerability and the delivery of planned treatment courses without dose reductions or delays. Two trials have now tested these principles prospectively, taking complementary approaches to integrating Lu-PSMA into first-line mHSPC treatment.

### 3.2. Concurrent Intensification with Standard of Care: PSMAddition

PSMAddition is an international, randomized Phase III trial evaluating Lu-PSMA plus standard of care (androgen receptor pathway inhibitor and ADT) versus standard of care alone in patients with metastatic hormone-sensitive prostate cancer (mHSPC). This represents a shift toward using radioligand therapy much earlier in the disease trajectory, at initial diagnosis of metastatic disease rather than after progression through multiple lines of therapy. The trial enrolled 1144 treatment-naïve or minimally treated patients with PSMA-positive disease, randomized 1:1 to receive either Lu-PSMA (7.4 GBq every 6 weeks for up to 6 cycles) plus an ARPI and ADT, or ARPI and ADT alone. The trial included patients with both high-volume and low-volume disease, and both de novo and metachronous presentations [13].

Top-line results presented at the 2025 Society of Urologic Oncology (SUO) annual meeting showed that at a median follow-up of 23.6 months, PSMAddition met its primary endpoint of improved rPFS (HR 0.72, 95% CI 0.58–0.90; *p* = 0.002). The benefit was consistent across prespecified subgroups, including high-volume vs. low-volume disease and de novo vs. metachronous presentation. Key secondary endpoints also favored the Lu-PSMA arm—the rate of undetectable PSA (<0.2 ng/mL) was 87% vs. 75%, the complete radiographic response rate was 57% vs. 42%, and the time to castration resistance was prolonged (HR 0.70, 95% CI 0.58–0.84). A trend toward improved overall survival was observed (HR 0.84, 95% CI 0.64–1.13; *p* = 0.125), though the data remains immature. No clinically significant differences in time to worsening in health-related quality of life and pain were observed, suggesting that the additional treatment burden of Lu-PSMA did not meaningfully impair patient well-being despite the addition of six infusion cycles. The safety profile was consistent with known toxicities of both Lu-PSMA and ARPIs. Grade 3 or higher adverse events occurred in 51% vs. 43% of patients, driven primarily by higher rates of cytopenias in the Lu-PSMA arm (grade ≥ 3 anemia 28% vs. 14%; neutropenia 15% vs. 4%; thrombocytopenia 11% vs. 3%). Dry mouth occurred in 46% vs. 4%, though was predominantly grade 1.

PSMAddition establishes proof of concept for intensifying first-line mHSPC treatment with radioligand therapy. However, several questions remain unanswered. OS data require further maturation before definitive conclusions about survival benefit can be drawn. Adding Lu-PSMA to an already-intensified ADT-plus-ARPI backbone means that the incremental benefit is being measured against an increasingly effective standard of care, which may attenuate absolute treatment effects. Additionally, the role of tumor volume, PSMA expression intensity, and molecular subtypes in predicting differential benefit requires further analysis.

### 3.3. Induction Therapy Before Chemotherapy: UpFrontPSMA

The UpFrontPSMA trial took a complementary approach, evaluating Lu-PSMA as an upfront induction therapy before docetaxel in de novo high-volume mHSPC. This Australian randomized Phase II trial assigned 130 patients to two cycles of Lu-PSMA (7.5 GBq every 6 weeks) followed by six cycles of docetaxel plus ADT, or docetaxel plus ADT alone. The primary endpoint was the rate of undetectable PSA (≤0.2 ng/mL) at 48 weeks, which was shown to be significantly higher in the Lu-PSMA arm (41% vs. 16%; odds ratio 3.88, 95% CI 1.61–9.38; *p* = 0.002). Lu-PSMA induction also improved PSA progression-free survival (31 vs. 20 months; HR 0.60, *p* = 0.039) and time to castration resistance (20 vs. 16 months; HR 0.60, *p* = 0.033). Radiographic PFS showed a trend toward improvement that did not reach statistical significance (HR 0.58, *p* = 0.067), and OS data remained immature at a median follow-up of 2.5 years [14].

Grade 3–4 treatment-related adverse events were comparable between arms (29% vs. 27%), with no treatment-related deaths. Dry mouth occurred in 37% of the Lu-PSMA arm (all grade 1), consistent with the known toxicity profile.

UpFrontPSMA demonstrates that Lu-PSMA can be safely integrated as induction therapy before chemotherapy in mHSPC, with enhanced biochemical responses. Importantly, it tests a different sequencing strategy from PSMAddition with Lu-PSMA administered before chemotherapy rather than concurrent with an ARPI. These complementary approaches will inform the optimal positioning of RLT within the increasingly complex mHSPC treatment algorithm.

## 4. Lu-PSMA in Oligometastatic/Oligorecurrent Disease

### 4.1. Lu-PSMA as Metastasis-Directed Therapy: BULLSEYE

The BULLSEYE trial was a randomized Phase II study that evaluated Lu-PSMA as metastasis-directed therapy (MDT) in oligometastatic hormone-sensitive prostate cancer [15]. The trial enrolled 58 patients who had biochemically recurrent prostate cancer following radical prostatectomy or radiotherapy, with up to 5 PSMA PET-positive metastases and PSA doubling time less than 6 months, who were randomized to receive either Lu-PSMA (two to four cycles of 7.4 GBq every 6 weeks) or standard of care (deferred ADT). At median follow-up of 7 months, median PFS was not reached in the Lu-PSMA group versus 5 months with deferred ADT (HR 0.07, 95% CI 0.02–0.19, *p* < 0.001). Median PSA change was −91% versus +125%, with 24% achieving complete biochemical remission. Median PSA-PFS was 17 months and median ADT-free survival was 26 months in treated patients. Treatment was well tolerated with predominantly grade 1 adverse events and rare grade 2 or higher toxicity. These early findings suggest that, in selected patients with oligometastatic HSPC, Lu-PSMA may allow deferral of first-line hormonal therapy, particularly for patients ineligible for focal therapies, and that receptor-targeted radionuclide therapies may potentially offer a way of salvaging low-volume cancers—conceptually analogous to the use of radioiodine in metastatic thyroid cancer. These conclusions are however constrained by the small sample size and short follow-up, and require further confirmation in larger trials.

### 4.2. Neoadjuvant Lu-PSMA Before Ablative Radiotherapy: LUNAR

In the same MDT space, the LUNAR (Lutetium-PSMA Neoadjuvant to Ablative Radiotherapy for Oligorecurrent Prostate Cancer) trial evaluated ^177^Lu-PNT2002 added to stereotactic body radiotherapy in oligorecurrent hormone-sensitive prostate cancer [16]. 87 evaluable patients with one to five PSMA PET-positive lesions were randomized to SBRT alone or two cycles of ^177^Lu-PNT2002 (6.8 GBq per cycle, 2 weeks apart) followed by SBRT. At median follow-up of 22 months, neoadjuvant ^177^Lu-PNT2002 significantly improved PFS (17.6 vs. 7.4 months; HR 0.37, 95% CI 0.22–0.61; *p* < 0.0001), representing a 63% reduction in progression risk. Salvage hormonal therapy-free survival was also prolonged (24.3 vs. 14.1 months; HR 0.40, *p* < 0.0001), and PSA50 response rates were higher (52% vs. 31%; *p* = 0.04). Of note, 98% of progression events were driven by new lesions on PSMA-PET rather than in-field recurrence, suggesting that the benefit of neoadjuvant Lu-PSMA derived from activity against occult micrometastatic disease that SBRT alone could not address. Toxicity was minimal, with only grade 3 lymphopenia observed (7% in the Lu-PSMA arm). No dose reductions or treatment discontinuations were required.

Together, BULLSEYE and LUNAR challenge the traditional paradigm of reserving systemic radionuclide therapy for advanced, heavily pretreated disease. Both trials demonstrate that Lu-PSMA can be deployed safely in patients with low-volume, hormone-sensitive disease, achieving meaningful disease control while deferring the initiation of ADT and its associated morbidity. Further trials are needed to confirm these findings, and to identify optimal patient selection criteria for this approach.

A summary of key clinical trials in PSMA-targeted radioligand therapy is provided in Table 1.

## 5. Next-Generation PSMA-Targeted RLT

Despite the clinical advances described above, lutetium-177 has inherent radiobiological limitations. Its beta emissions have a relatively long path length (~670 μm) that may undertreat isolated tumor cells and micrometastatic deposits, and a proportion of patients either do not respond or eventually progress. These limitations have motivated the development of alternative radionuclides with distinct physical properties, including alpha-emitters with higher linear energy transfer and Auger electron emitters with nanometer-range cytotoxicity [17,18].

### 5.1. Alpha-Emitter Therapy with Actinium-225: WARMTH

The WARMTH (Worldwide Actinium-225-PSMA Radioligand Therapy of Metastatic Castration-Resistant Prostate Cancer) study was an international, multicenter retrospective analysis evaluating the clinical utility of alpha-emitting radioligand therapy in the late-line setting [19]. This study cohort comprised 488 patients with heavily pretreated mCRPC who underwent treatment with ^225^Ac-PSMA between 2016 and 2023. The study population was characterized by extensive prior therapeutic exposure, including docetaxel (66%), abiraterone (39%), and enzalutamide (39%); notably, 32% of patients had progressed following Lu-PSMA, providing clinical evidence for the efficacy of alpha-emitters in beta-refractory disease. Efficacy outcomes were encouraging, with a median overall survival of 15.5 months, median progression-free survival of 7.9 months, and substantial PSA50 response rates. Biologically, the high linear energy transfer of ^225^Ac-targeted alpha therapy offers superior cytotoxic potency compared to beta-particle radiation, facilitating tumor cell death via double-strand DNA breaks with fewer direct hits. The toxicity profile was dominated by xerostomia, which was reported in 68% of patients after the initial cycle and occurred universally with prolonged treatment (greater than seven cycles). Grade 3 or higher hematologic toxicities were observed in a minority of patients (anemia, 13%; thrombocytopenia, 7%; leukopenia, 4%), while grade 3–4 renal toxicity was limited to 5%, with no reported treatment-related mortality.

### 5.2. Dual Beta-Auger Therapy with Terbium-161: VIOLET

The novel radioligand Terbium-161 (^161^Tb-PSMA) is a dual beta-Auger emitter which provides beta radiation comparable to ^177^Lu, in addition to an abundant emission of Auger and conversion electrons. These particles possess ultra-short path lengths that potentially facilitate superior targeting of micrometastatic deposits and individual tumor cells that may remain “energy-sheltered” from the longer-range emissions of ^177^Lu. The VIOLET trial, a first-in-human, single-arm Phase I/II investigation, evaluated the clinical utility of ^161^Tb-PSMA in patients with progressive mCRPC following exposure to ARPIs and taxane chemotherapy [20]. Efficacy outcomes in this small cohort of 30 patients were encouraging, with a PSA50 response rate of 70% (95% CI 51–85%) and a PSA90 response rate of 40% (95% CI 23–59%). In this heavily pretreated population, the median PSA-PFS was 9.0 months and median rPFS reached 11.1 months. The safety profile remained favorable; grade 3 or higher adverse events were limited to lymphopenia and transient pain flares, with no reported treatment-related mortality.

### 5.3. Emerging Radionuclides and Tandem Strategies

Beyond actinium-225 and terbium-161, a diverse array of alpha and beta-emitting radioisotopes are under clinical and preclinical investigation for PSMA-targeted radioligand therapy. Alpha-emitters currently in development include thorium-227 ([^227^Th]Th-BAY2315497), lead-212 ([^212^Pb]Pb-ADVC001), and astatine-211, all characterized by high linear energy transfer (LET) and short path lengths (<100 μm) that cause cytotoxic double-stranded DNA breaks whilst minimizing crossfire radiation [21,22]. Beta-emitters under investigation include iodine-131 ([^131^I]I-1095) and copper-67 ([^67^Cu]Cu-SAR-bisPSMA), each offering different emission energies and half-lives that may be optimized for specific clinical scenarios [23,24]. An emerging strategy involves tandem or combination therapies that leverage complementary properties of alpha and beta-emitters. The AlphaBet trial evaluated combining Lu-PSMA with radium-223 in 37 mCRPC patients with bone metastases, demonstrating safety and feasibility. PSA50 response was observed in 18 (55%; 95% CI 36–72) patients at a median follow-up of 13.3 months [25].

A summary of next-generation PSMA-targeted radionuclides that are currently being studied is provided in Table 2.

**Figure 1:**
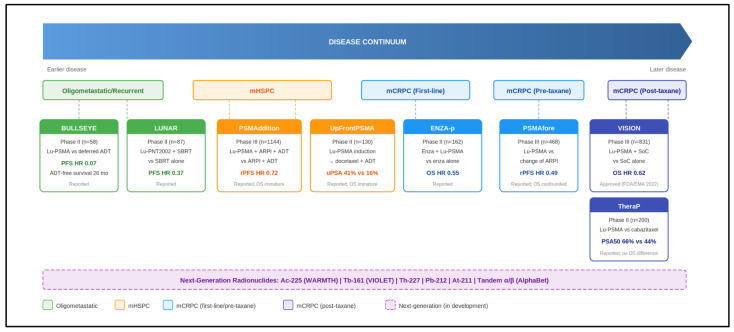
PSMA-targeted radioligand therapy across the prostate cancer disease continuum.

Figure 1 illustrates the key clinical trials in PSMA-targeted radioligand therapy in relation to the prostate cancer disease spectrum.

## 6. Future Directions

### 6.1. Patient Selection

As PSMA-targeted RLT moves into earlier disease settings, robust patient selection becomes increasingly important. The current standard of PSMA-PET positivity is necessary, but likely insufficient. Analyses of VISION-eligible patients treated with Lu-PSMA demonstrated that those who would have been TheraP-ineligible due to discordant disease (FDG-avid but PSMA-negative lesions) have significantly poorer outcomes [26]. Dual-tracer imaging with PSMA-PET and ^18^F-FDG-PET, as employed for eligibility in the TheraP and LuPSMA trials, can identify FDG-avid, PSMA-negative disease that is unlikely to respond to radioligand therapy and may instead warrant alternative treatment, such as directed local treatment [27]. Quantitative PSMA-PET metrics, including SUVmean and tumor volume, are being investigated as predictive biomarkers, though their optimal thresholds and measurement standardization remain undefined [28].

Liquid biopsy analysis is an innovative technology which may complement imaging-based selection for PSMA-targeted RLT. Circulating tumor cell (CTC) PSMA expression can capture tumor heterogeneity not fully reflected by imaging, though the evidence base remains limited [29]. Alternatively, circulating tumor DNA (ctDNA) analysis shows more robust predictive potential, with low ctDNA fraction predicting superior biochemical response and progression-free survival independent of PSMA-PET parameters [30]. Specific genomic alterations detected in ctDNA, including FGFR1 and CCNE1 amplifications, CDK12 mutations, and markers of genomic instability, have been associated with resistance to Lu-PSMA therapy in exploratory analyses [31,32]. The integration of liquid biopsy and imaging biomarkers into composite selection tools remains investigational, with current evidence consisting primarily of exploratory analyses in small cohorts requiring validation before clinical implementation.

### 6.2. Treatment Sequencing and Integration

The optimal sequencing of Lu-PSMA relative to hormonal therapies and chemotherapy remains uncertain. PSMAddition tests concurrent Lu-PSMA with ADT and ARPI; UpFrontPSMA tests sequential Lu-PSMA before chemotherapy. Whether upfront induction, concurrent combination, or maintenance strategies yield the best long-term outcomes will require additional randomized data. In addition, newer combinations, such as with Proteolysis Targeting Chimeras (PROTACs) are being explored. LuxAR-02 (NCT07047118) is a Phase II randomized study evaluating luxdegalutamide, an oral PROTAC androgen receptor degrader targeting wild-type and mutant AR, in combination with Lu-PSMA versus Lu-PSMA alone in patients with PSMA-positive mCRPC after prior ARPI and up to two taxanes [33]. The rationale rests on PROTAC-mediated AR degradation addressing a key resistance pathway alongside PSMA-targeted radionuclide cytotoxicity. Enrollment is ongoing, with preliminary results awaited.

### 6.3. Access, Infrastructure, and Equity

The expansion of Lu-PSMA into earlier disease settings has significant implications for healthcare infrastructure and equity. PSMA-PET/CT for patient selection, radio-pharmacy capacity, nuclear medicine facilities for RLT administration, and radiation safety infrastructure are not uniformly available globally [34,35], with reimbursement protocols and established referral pathways even less so. In the Asia-Pacific region, where prostate cancer incidence is rising rapidly and healthcare systems face competing resource demands, the gap between clinical evidence and treatment access is particularly pronounced [36]. Cost-effectiveness data for Lu-PSMA in earlier disease settings such as mHSPC are currently absent, though analyses in mCRPC show variable cost-effectiveness depending on healthcare system, comparator, and assumptions [37,38,39]. The incremental cost of adding RLT to already-intensified regimens will require formal health economic evaluation before adoption in resource-sensitive systems. Collaborative regional efforts to establish PSMA-PET and RLT infrastructure, coupled with context-specific cost-effectiveness analyses, will be essential to ensure equitable access.

## 7. Conclusions

The evidence base for PSMA-targeted radioligand therapy has expanded substantially beyond the post-taxane mCRPC setting in which it was first approved. In mCRPC, the progression from VISION and TheraP through PSMAfore to ENZA-p has demonstrated benefit across an increasingly broad patient population and established the principle of combining RLT with hormonal therapy. PSMAddition and UpFrontPSMA now extend this trajectory into mHSPC, testing concurrent intensification and upfront induction strategies, respectively, in a population characterized by higher PSMA expression and greater treatment tolerability. In oligometastatic disease, BULLSEYE and LUNAR provide early evidence that Lu-PSMA may function as metastasis-directed therapy with the potential of deferring ADT and its associated long-term morbidity. Next-generation radionuclides, including actinium-225 and terbium-161, offer distinct radiobiological properties that may address the limitations of beta-emitter therapy, particularly in micrometastatic disease and after lutetium-177 progression.

Several important caveats apply. Overall survival data for the mHSPC and oligometastatic trials remain immature, and the incremental benefit of adding RLT to already-intensified standard-of-care regimens may prove modest in absolute terms. The acceptable toxicity threshold also shifts as treatment moves earlier—xerostomia and cytopenias that are tolerable in heavily pre-treated mCRPC carry greater weight in hormone-sensitive disease, where survival is longer and effective standard-of-care alternatives exist. Patient selection beyond binary PSMA-PET positivity requires refinement; quantitative imaging metrics, ctDNA-based biomarkers, and dual-tracer approaches are all under investigation but none are validated for routine clinical use. Infrastructure for PSMA-PET and RLT administration remains unevenly distributed globally, and cost-effectiveness in earlier disease settings has not been formally evaluated, a gap that is particularly relevant in the Asia-Pacific region and other resource-sensitive healthcare systems.

The direction of travel is nonetheless clear. PSMA-targeted RLT is moving from a late-line option in refractory mCRPC toward potential incorporation into first-line treatment of hormone-sensitive disease. Realizing this potential will depend on confirmatory survival data from ongoing trials, the development of validated biomarker-driven patient selection, and the establishment of accessible and cost-effective treatment pathways across diverse healthcare systems.

## Figures and Tables

**Table 1:** Summary of key clinical trials in PSMA-targeted radioligand therapy.

Trial	Phase	Setting	N	Intervention vs. Comparator	Primary Endpoint	Key Efficacy Results	OS Data	Evidence Level	Key Safety Findings
VISION [3]	III	mCRPC, post-taxane	831	Lu-PSMA (7.4 GBq Q6W, 4–6 cycles) + SoC vs. SoC alone	rPFS and OS (co-primary)	rPFS: 8.7 vs. 3.4 mo (HR 0.40, 99.2% CI 0.29–0.57); OS: 15.3 vs. 11.3 mo (HR 0.62, 95% CI 0.52–0.74)	Mature– significant OS benefit (HR 0.62; *p* < 0.001)	Phase III; regulatory-grade, practice-defining	Dry mouth 39%; Gr ≥ 3 anemia 13%, thrombocytopenia 8%, lymphopenia 8%
TheraP [7]	II	mCRPC, post-docetaxel	200	Lu-PSMA (6.0–8.5 GBq Q6W, ≤6 cycles) vs. cabazitaxel	PSA50 response rate	PSA50: 66% vs. 44% (*p* = 0.0016); PFS RMST: 7.1 vs. 5.0 mo (*p* = 0.005)	Mature—no significant difference (not powered for OS)	Randomised Phase II vs. active comparator	Gr 3–4 AEs 33% vs. 53%; favorable toxicity profile vs. cabazitaxel
PSMAfore [9]	III	mCRPC, taxane-naive	468	Lu-PSMA (7.4 GBq, ≤6 cycles) vs. change of ARPI	rPFS	rPFS: 11.6 vs. 5.6 mo (HR 0.49, 95% CI 0.39–0.61)	Mature—ITT no significant difference (HR 0.91), crossover-adjusted HR 0.59	Phase III; rPFS practice-informing, OS interpretation limited	Improved QoL and pain outcomes vs. ARPI change
ENZA-p [12]	II	mCRPC, first-line	162	Enzalutamide + Lu-PSMA (adaptive 2–4 cycles) vs. enzalutamide alone	PSA-PFS	PSA-PFS: 13.0 vs. 7.8 mo (HR 0.43; *p* < 0.0001)	Mature—significant OS benefit (HR 0.55, 95% CI 0.36–0.84); secondary endpoint	Randomised Phase II; OS benefit hypothesis-generating	Adaptive dosing guided by PSMA-PET response; manageable toxicity
PSMAddition [13]	III	mHSPC, first-line	1144	Lu-PSMA (7.4 GBq Q6W, ≤6 cycles) + ARPI + ADT vs. ARPI + ADT	rPFS	rPFS: HR 0.72 (95% CI 0.58–0.90; *p* = 0.002); uPSA 87% vs. 75%; cRR 57% vs. 42%	Immature—trend towards OS benefit (HR 0.84 (95% CI 0.64–1.13)	Phase III; primary endpoint met, OS pending; practice-informing	Gr ≥3 AEs 51% vs. 43%; Gr ≥ 3 anemia 28% vs. 14%; dry mouth 46% vs. 4%
UpFrontPSMA [14]	II	mHSPC, de novo high-volume	130	Lu-PSMA (7.5 GBq Q6W, 2 cycles) → docetaxel + ADT vs. docetaxel + ADT	Undetectable PSA at 48 wk	uPSA at 48 wk: 41% vs. 16% (OR 3.88; *p* = 0.002)	Immature	Randomised Phase II; signal-seeking	Gr 3–4 AEs comparable (29% vs. 27%); no treatment-related deaths; dry mouth 37% (all Gr 1)
BULLSEYE [15]	II	Oligometastatic HSPC	58	Lu-PSMA (7.4 GBq Q6W, 2–4 cycles) vs. deferred ADT	PFS	PFS: NR vs. 5 mo (HR 0.07, 95% CI 0.02–0.19; *p* < 0.001); PSA change −91% vs. +125%	Not reported	Small randomised Phase II (*n* = 58); hypothesis-generating	Predominantly Gr 1 AEs; rare Gr ≥ 2 toxicity
LUNAR [16]	II	Oligorecurrent HSPC	87	^177^Lu-PNT2002 (6.8 GBq, 2 cycles) + SBRT vs. SBRT alone	PFS	PFS: 17.6 vs. 7.4 mo (HR 0.37, 95% CI 0.22–0.61; *p* < 0.0001); PSA50: 52% vs. 31%	Not reported	Randomised Phase II; hypothesis-generating	Minimal; Gr 3 lymphopenia 7%; no dose reductions or discontinuations

ADT, androgen deprivation therapy; AE, adverse event; ARPI, androgen receptor pathway inhibitor; CI, confidence interval; cRR, complete radiographic response; Gr, grade; HR, hazard ratio; ITT, intention-to-treat; mCRPC, metastatic castration-resistant prostate cancer; mHSPC, metastatic hormone-sensitive prostate cancer; mo, months; NR, not reached; OR, odds ratio; OS, overall survival; PFS, progression-free survival; PSA50, ≥50% PSA decline; Q6W, every 6 weeks; RMST, restricted mean survival time; rPFS, radiographic PFS; SBRT, stereotactic body radiotherapy; SoC, standard of care; uPSA, undetectable PSA; wk, weeks; yr, years.

**Table 2:** Summary of emerging next-generation PSMA-targeted radionuclides.

Radionuclide	Emission/Physical Characteristics	Development Stage	Current Clinical Evidence	Key Limitations
^177^Lu	β^−^-emitter; path length ~670 µm (reference standard)	FDA-approved (mCRPC)	VISION, TheraP, PSMAfore, ENZA-p; established efficacy	Long β path may undertreat micrometastases; primary/acquired resistance
^225^Ac	α-emitter; high LET, short range with path length 40–100 µm	Clinical—retrospective; prospective trials ongoing	WARMTH (*n* = 488): median OS 15.5 mo, median PFS 7.9 mo; active in β-refractory disease	Xerostomia (universal beyond ~7 cycles); daughter redistribution effect; global scarcity
^161^Tb	Dual β^−^–Auger emitter; β comparable to ^177^Lu plus short-range electrons	Early clinical; Phase I	VIOLET (*n* = 30): PSA50 70%, rPFS 11.1 mo	Very small single-arm cohort; production & supply constraints
^227^Th	α-emitter; high LET, path 20–100 µm	Early clinical; Phase I	BAY2315497; no reported efficacy outcomes yet	Early stage investigational status; chelation and toxicity data immature
^212^Pb	β^−^ then daughter decay α-emitter; path length 50–80 µm	Early clinical; Phase I	TheraPb; no reported efficacy outcomes yet	Short half-life limits logistics; possible nephrotoxicity
^211^At	α-emitter; path length 50–90 µm	Preclinical	Studies still ex vivo	Production constraints; short half-life
^131^I	β^−^-emitter (with γ emission); path length 0.4–2.4mm	Early clinical; Phase II	ARROW (*n* = 120); PSA50 62.9%, rPFS 14 mo	Xerostomia; γ emission and radiation-safety considerations
^67^Cu	β^−^-emitter; theranostic pairing	Early clinical; Phase I	SAR-bisPSMA; no reported efficacy outcomes yet	Isotope availability and production scale
^177^Lu + ^223^Ra	Tandem β^−^ (PSMA) + α (bone-targeting)	Early clinical; Phase I/II single-arm	AlphaBet (*n* = 37): PSA50 55%; safe and feasible	Small, single-centre; feasibility/safety focus only

## Data Availability

No new data were created or analyzed in this study. Data sharing is not applicable to this article.

## Abbreviations

The following abbreviations are used in this manuscript:

Lu-PSMA	Lutetium-177-PSMA-617
mCRPC	Metastatic castration-resistant prostate cancer
mHSPC	Metastatic hormone-sensitive prostate cancer
ADT	Androgen deprivation therapy
ARPI	Androgen receptor pathway inhibitor
PSMA	Prostate-specific membrane antigen
RLT	Radioligand therapy
RMST	Restricted mean survival time
rPFS	Radiographic progression-free survival
OS	Overall survival
